# Glomerular mTORC1 activation was associated with podocytes to endothelial cells communication in lupus nephritis

**DOI:** 10.1136/lupus-2023-000896

**Published:** 2023-05-05

**Authors:** Xiaotian Liu, Zhaomin Mao, Mo Yuan, Linlin Li, Ying Tan, Zhen Qu, Min Chen, Feng Yu

**Affiliations:** 1 Renal Division, Peking University First Hospital, Beijing, China; 2 Institute of Nephrology, Peking University, Beijing, China; 3 Key Laboratory of Renal Disease, Ministry of Health of China, Beijing, China; 4 Key Laboratory of CKD Prevention and Treatment, Ministry of Education of China, Beijing, China; 5 Research Units of Diagnosis and Treatment of lmmune-Mediated Kidney Diseases, Chinese Academy of Medical Sciences, Beijing, China; 6 Department of Nephrology, Peking University International Hospital, Beijing, China

**Keywords:** lupus nephritis, autoimmune diseases, inflammation

## Abstract

**Objective:**

This study was initiated to evaluate the mammalian target of the rapamycin (mTOR) signalling pathway involved in renal endothelial-podocyte crosstalk in patients with lupus nephritis (LN).

**Methods:**

We compared the kidney protein expression patterns of 10 patients with LN with severe endothelial-podocyte injury and 3 patients with non-severe endothelial-podocyte injury on formalin-fixed paraffin-embedded kidney tissues using label-free liquid chromatography-mass spectrometry for quantitative proteomics analysis. Podocyte injury was graded by foot process width (FPW). The severe group was referred to patients with both glomerular endocapillary hypercellularity and FPW >1240 nm. The non-severe group included patients with normal endothelial capillaries and FPW in the range of 619~1240 nm. Gene Ontology (GO) enrichment analyses were performed based on the protein intensity levels of differentially expressed proteins in each patient. An enriched mTOR pathway was selected, and the activation of mTOR complexes in renal biopsied specimens was further verified in 176 patients with LN.

**Results:**

Compared with those of the non-severe group, 230 proteins were upregulated and 54 proteins were downregulated in the severe group. Furthermore, GO enrichment analysis showed enrichment in the ‘positive regulation of mTOR signalling’ pathway. The glomerular activation of mTOR complex 1 (mTORC1) was significantly increased in the severe group compared with the non-severe group (p=0.034), and mTORC1 was located in podocytes and glomerular endothelial cells. Glomerular activation of mTORC1 was positively correlated with endocapillary hypercellularity (r=0.289, p<0.001) and significantly increased in patients with both endocapillary hypercellularity and FPW >1240 nm (p<0.001).

**Conclusions:**

Glomerular mTORC1 was highly activated in patients with both glomerular endocapillary hypercellularity and podocyte injury, which might be involved in podocytes to endothelial cells communication in lupus nephritis.

**WHAT IS ALREADY KNOWN ON THIS TOPIC**

- Endothelial cell-podocyte crosstalk might play a critical role in glomerular injury in lupus nephritis, and its regulatory molecular mechanisms still need to be explored.

**WHAT THIS STUDY ADDS**

- Glomerular mammalian target of the rapamycin (mTOR) complex 1 activation might be involved in podocytes to endothelial cells communication in lupus nephritis.

**HOW THIS STUDY MIGHT AFFECT RESEARCH, PRACTICE OR POLICY**

- Rational inhibition of mTOR after evaluating the renal activation of mTOR may especially help rescue glomerular injury.

## Introduction

SLE is a chronic autoimmune disease that affects multiple organs, and the kidneys are involved in nearly 60% of patients.^1 2^ Glomeruli are the main target of inflammation and immune deposits in lupus nephritis (LN).^2 3^ Both the 2018 International Society of Nephrology/Renal Pathology Society (ISN/RPS) classification for LN and National Institutes of Health (NIH) activity and chronicity indices highlighted the clinical value of glomerular lesions in LN.^3^

Our previous studies suggested that glomerular endothelial cells and podocyte injury were both prominent lesions in LN.^4–7^ In particular, the loss of podocyte integrity measured by foot process width (FPW) was positively correlated with the level of proteinuria, and a threshold FPW >1240 nm was identified to differentiate nephrotic proteinuria from non-nephrotic proteinuria in LN.^6^ More importantly, the renal pathological scores of endothelial cell swelling and/or proliferation were positively correlated with FPW in patients with LN complicated with thrombotic microangiopathy (TMA).^7^ The vascular endothelial growth factor (VEGF) and endothelin-1 system between glomerular endothelial cells and podocytes might play a critical role in the association between endothelial and podocyte injury,^7–11^ although a more precise molecular signalling pathway concerning their crosstalk remains to be further elucidated.

Mammalian target of rapamycin (mTOR) is an evolutionarily conserved serine-threonine kinase that regulates cell growth, proliferation, metabolism and survival in response to hormonal and nutrient signals.^12–15^ Increasing evidence indicates that mTOR plays an important role in the regulation of renal cell homeostasis and autophagy.^14–16^ More interestingly, our previous work showed that high glomerular activation of mTOR complex 1 and 2 (mTORC1/2) was observed in endothelial cells and podocytes in patients with LN.^17^ The activation of glomerular mTORC1 was especially associated with pathological endocapillary hypercellularity and clinical proteinuria.^17^ Thus, we propose that mTOR signalling pathways may be involved in the endothelial-podocyte crosstalk in LN.

Here, we initially evaluated the alterations in protein expression profiles based on proteomics in the renal specimens of patients with LN with different degrees of endothelial cell and podocyte injury, and intended to uncover the role of mTOR signalling pathways in the endothelial-podocyte crosstalk of the disease with larger samples.

## Materials and methods

### Patients

**Figure 1:**
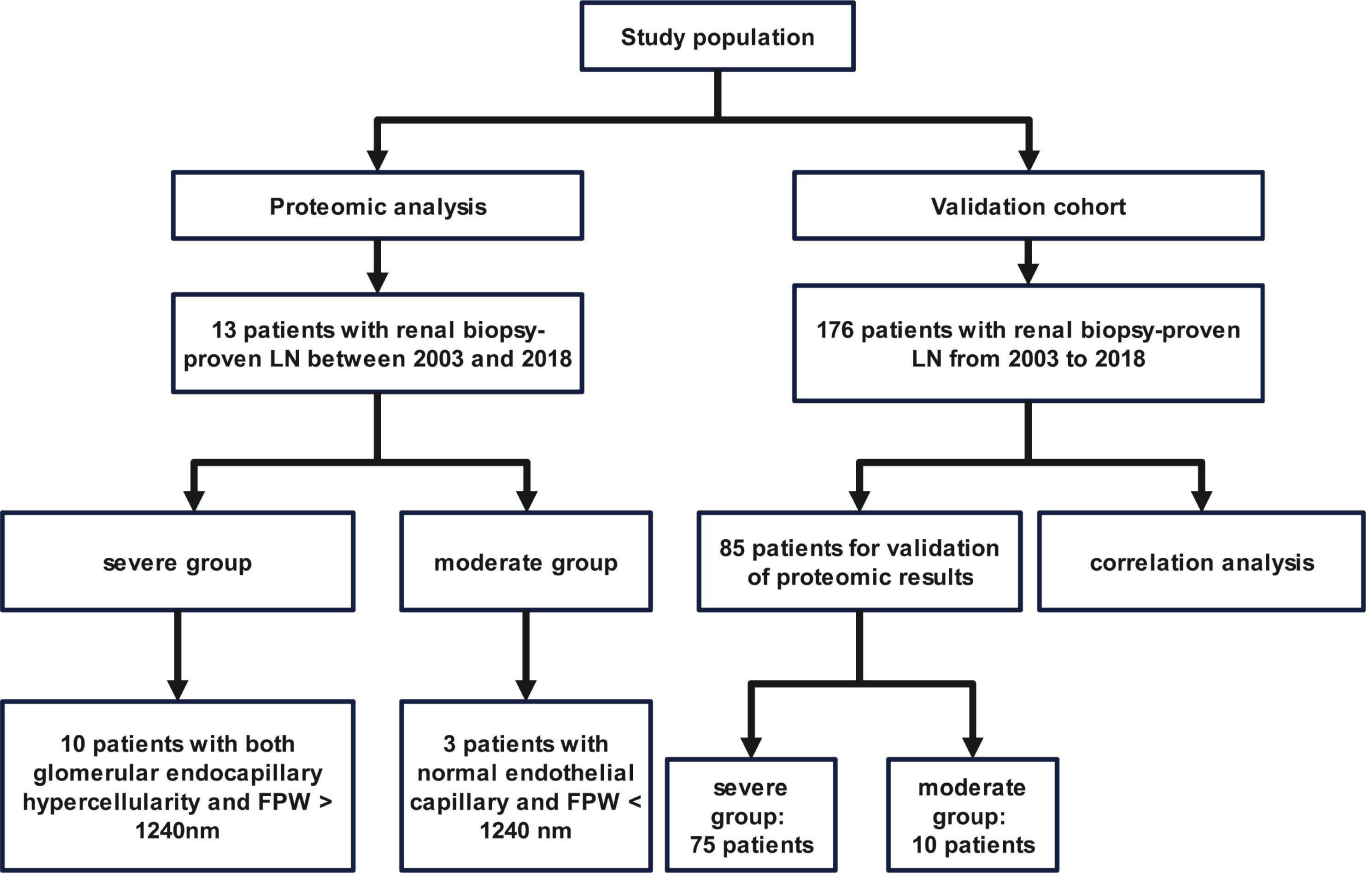
Flow chart of the enrolled population. FPW, foot process width; LN, lupus nephritis.

Complete clinical and pathological data from 13 patients with renal biopsy-proven LN for proteomic analysis (the baseline data are listed in online supplemental table 1) and 176 patients with renal biopsy-proven LN for further verified analysis (their baseline data are listed in online supplemental table 2) at Peking University First Hospital from 2003 to 2018 were collected. Among the 176 patients, 75 in the severe endothelial-podocyte group and 10 in the non-severe endothelial-podocyte group were selected for validation of the proteomic results (figure 1). To investigate the specificity of our findings, we selected 17 patients with IgA nephropathy (IgAN) as a control group: 10 patients who had endocapillary proliferation (according to the 2016 Oxford Classification of IgA nephropathy^18^) and 7 patients without endocapillary proliferation.

10.1136/lupus-2023-000896.supp1 Supplementary data

### Clinical evaluation

The clinical data of patients with LN were extracted from the electronic medical records of Peking University First Hospital. The disease activity was assessed by the SLE Disease Activity Index (SLEDAI).^19 20^ Serum ANAs and anti-double-stranded DNA antibodies were detected using an indirect commercial immunofluorescence assay. Serum C3 was determined using a rate nephelometry assay (Beckman-Coulter, IMMAGE, Fullerton, California, USA).

### Renal histopathology

Renal biopsy specimens were examined according to the 2018 ISN/RPS classification system^3^ by light microscopy, direct immunofluorescence and electron microscopy. Pathological parameters, including activity indices (AI) and chronicity indices (CI), were determined by semi-quantitative scoring of specific biopsy features.^3^ The semi-quantification of glomerular endothelial cell injury was referred to as endocapillary hypercellularity defined by pathologists based on the NIH system.^3^

### Morphometric analysis of FPW

Morphometric analysis of FPW was performed as described previously.^6 7^ From each patient, the arithmetic mean of the FPW was calculated as follows:

$$FPW=\frac{\pi }{4}\times \frac{\sum GBM\;length}{\sum foot\;process}$$

Podocyte injury was graded by FPW, and FPW >1240 nm was the most applicable cut-off value that could differentiate nephrotic proteinuria from non-nephrotic proteinuria with a sensitivity of 81.5% and a specificity of 62.7% by ROC curve analysis in patients with LN according to our previous data.^6 7^ In our centre, the normal range of FPW was 553±34 nm.^6^ The bilateral reference range of normal distribution data was usually the 95% central of normal results, which was calculated as ‘$\bar{X}\pm 1.96S$’ ($\bar{X}=553$, S=34); thus, the reference range of FPW in healthy people was 486~619 nm. Severe podocyte injury was defined as an FPW >1240 nm, and the non-severe group was defined based on an FPW in the range of 619~1240 nm.

### Mass spectrometry and proteomics

Formalin-fixed paraffin-embedded kidney tissues from patients with LN were digested by LysC and trypsin. After quantification of peptide concentration by Pierce Quantitative Colorimetric Peptide Assay kits, samples (2 mg each) were loaded for MS analysis. Next, label-free liquid chromatography-mass spectrometry analysis was performed on an Easy-nLC System (Thermo Fisher Scientific), and samples were analysed with a Q Exactive mass spectrometer (Thermo Fisher Scientific). The detailed mass spectrometry procedures were described in a previous study.^17 21^ Raw data were searched against the UniProt Homo species database. Proteins that met the inclusion criteria had at least a twofold change, and p<0.05 was used to identify differentially expressed proteins (DEPs). GO (Gene Ontology) enrichment analyses were performed on the David website (https://david.ncifcrf.gov/).

### Renal immunohistochemistry assay

Tissue samples were dewaxed and rehydrated. After antigen heated retrieval and blocking with 3% bovine serum albumin (BSA), tissues were incubated with primary rabbit antiphospho-S6 ribosomal protein (Ser 235/236) antibody or p-AKT (Ser473) (Cell Signaling Technology, Massachusetts, USA) (representative activation marker of mTORC1 and mTORC2, respectively)^17 22^ or anti-CD8 (Abcam) or anti-FOXP3 antibody (Sigma), followed by incubation with secondary antibody (ZSGB-Bio, PV9001) and colouration with 3,3-diaminobenzidine. For blank controls, primary antibodies were replaced by phosphate-buffered saline (PBS). Pararenal carcinoma tissue was collected as the negative control. Cell nuclei were stained with H&E. Image-Pro Plus analysis software (V.6.0; Media Cybernetics, Dallas, Texas, USA) was used to measure the mean optical density (integrated option density/area) in renal glomeruli and tubular interstitium.

### Renal immunofluorescence staining

Fresh frozen sections were blocked with 3% BSA, and then, rabbit antiphospho-S6 ribosomal protein (Ser 235/236) antibody (Cell Signaling Technology), combined with mouse antihuman CD31 (Santa Cruz) or goat antihuman synaptopodin (Santa Cruz), rabbit antihuman CD3 (Abcam) combined with mouse antihuman CD4 and CD8 (Abcam) and interleukin (IL)-17A conjugated with PE (BD Biosciences) were added and incubated overnight at 4°C, followed by the secondary antibodies Alexa Fluor 488-labelled donkey antirabbit IgG, Alexa Fluor 647-labelled donkey antimouse IgG (Abcam) or TRITC-labelled donkey antigoat IgG (Invitrogen) for 30 min at 37°C. Nuclei were stained with 4′,6-diamidino-2-phenylindole (ZSGB-Bio). For negative controls, primary antibodies were replaced by PBS. Fluorescence images were acquired with fluorescence microscopy (DM2500; Leica, Germany).

### Statistical analysis

Statistical software SPSS V.25.0 (SPSS, Chicago, Illinois, USA) and Prism V.9.0 software (GraphPad, San Diego, California, USA) were used for statistical analysis. The data are presented as the mean±SD for normally distributed data or median (IQR) for non-normally distributed data, and categorical data are expressed as numbers and ratios. Differences between groups of normally distributed data were assessed using t-tests, and non-normally distributed data were assessed using non-parametric tests. Reliability analysis was carried out to test the intraclass correlation coefficient between two observers. P values <0.05 were considered statistically significant.

## Results

### Differential proteome analysis of renal samples from patients with LN

**Figure 2:**
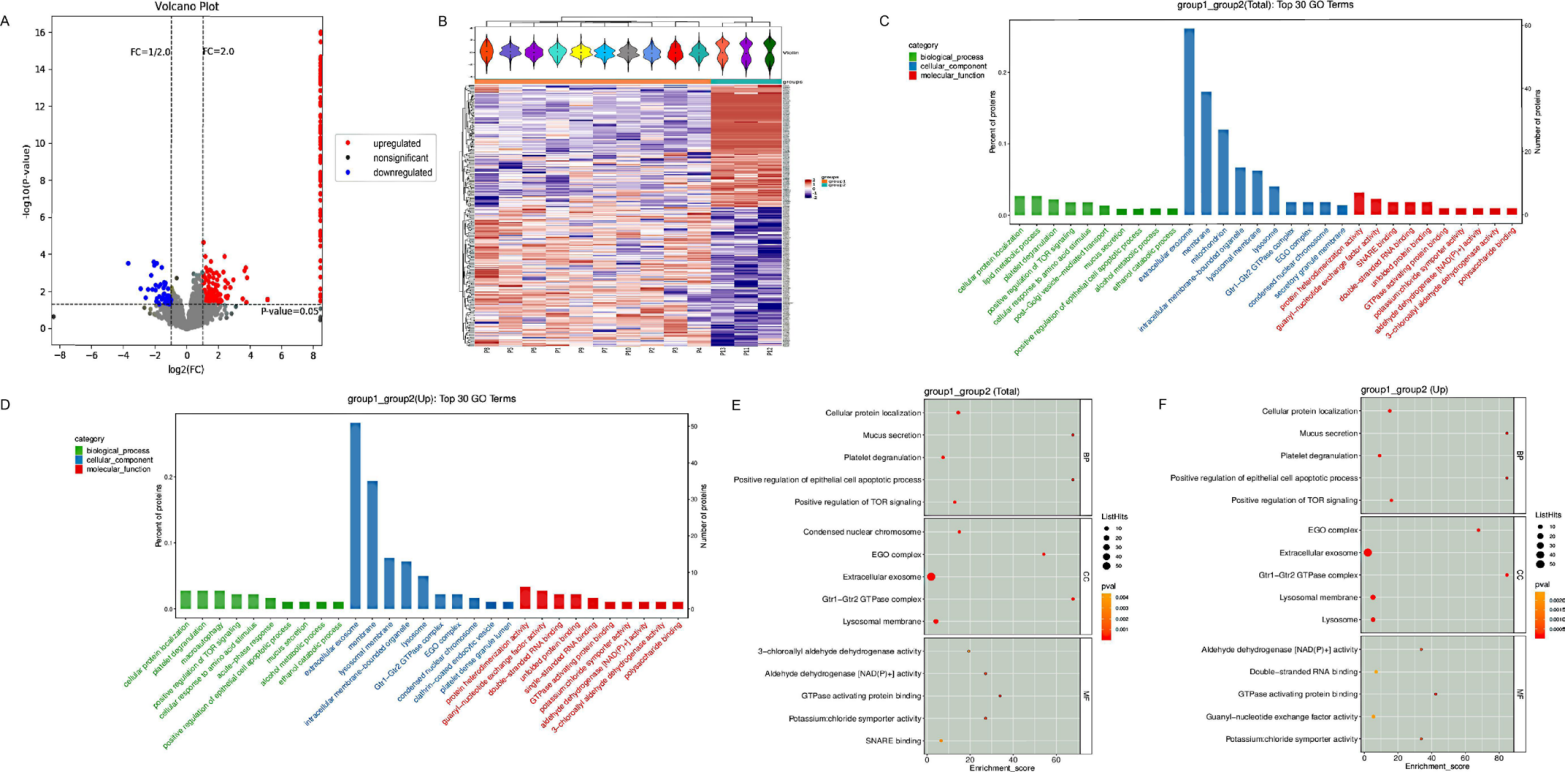
Quantitative proteomic and bioinformatic analyses of the renal specimens of patients with LN with severe and non-severe groups. (A) The volcano map depicts DEPs between the two groups. Red dots: upregulated proteins; green dots: downregulated proteins; grey dots: nonsignificant dots. (B) The heat maps demonstrated a clear difference in protein abundance levels across the two groups. Red bars: upregulated proteins; blue bars: downregulated proteins; green bars: sample P1-P10, non-severe group; yellow bars: sample P11-P13, severe group. (C) GO analysis items among total DEPs proteins. Green bars: biological process; blue bars: cellular complements; red bars: molecular function. (D) GO analysis items among upregulated DEPs proteins. Green bars: biological process; blue bars: cellular complements; red bars: molecular function. (E) The top five GO analysis items among total DEPs proteins. (F) The top five GO analysis items among total DEPs proteins. Group 1: severe group; group 2: non-severe group. BP, biological processes; CC, cellular components; DEP, differentially expressed protein; GO, Gene Ontology; LN, lupus nephritis; MF, molecular functions.

The 13 patients with LN were divided into two groups based on the degree of endothelial cell and podocyte injuries: the severe group (10 patients with both glomerular endocapillary hypercellularity and FPW >1240 nm) and the non-severe group (3 patients with normal endothelial capillaries and FPW <1240 nm). In total, 4700 credible proteins were detected by label-free quantitative proteomic analysis, with twofold change and p≤0.05 as the differential screening conditions. Finally, 284 DEPs were selected between the severe and non-severe groups. Data are shown in online supplemental table 3. The volcano diagram suggested that 230 proteins were upregulated and 54 proteins were downregulated in the severe group compared with the non-severe group (figure 2A). The heatmaps obtained from the analysis demonstrated a clear difference in protein abundance levels across the two groups (figure 2B).

### GO enrichment analysis

GO analysis was performed to enrich and cluster the DEPs of the severe group and non-severe group. Detailed information on the molecular functions, cellular components and biological processes is shown in figure 2C–D. GO annotation analysis revealed that the DEPs of the two groups were primarily involved in biological processes, including ‘cellular protein localisation’ (p<0.001), ‘platelet degranulation’ (p<0.001), ‘mucus secretion’ (p<0.001), ‘positive regulation of mTOR signalling’ (p<0.001) and ‘positive regulation of epithelial cell apoptotic process’ (p<0.001) (figure 2E–F). More interestingly, Ras-related GTPase (RRAG) A, RRAGB, RRAGC and RRAGD identified in the most prominently enriched pathway, ‘cellular protein localisation’ in GO enrichment analysis, were also covered in the biological process of ‘positive regulation of mTOR signalling’ (online supplemental table 4). RRAGs were demonstrated to necessarily recruit the mTOR complex to lysosomes to regulate cell growth and proliferation in response to hormonal and nutrient signals.^12 23^ Thus, the positive regulation of mTOR signalling might be associated with endothelial and podocyte injuries in LN.

### Validation of proteomic analysis in renal biopsied specimens of patients with LN

As mTOR signalling was found to be the most attractive pathway based on the above proteomic analysis, 85 patients with LN, 75 in the severe group and 10 in the non-severe group were selected for further validation.

Patients in the severe group presented with higher SLEDAI (p=0.049), proteinuria amount (p<0.001), serum creatinine value (p=0.010), pathological AI score (p<0.001) and CI score (p=0.043) than those in the non-severe group (table 1).

**Table 1:** Comparison of clinicopathological data between patients with LN in severe and non-severe group

	Severe (n=75)	Non-severe (n=10)	P value
Gender (male/female)	62/75	10/0	0.348
Age (years)	31 (25–40)	28 (25–38)	0.623
Proteinuria amount (g/24 hours)	3.98 (2.08–5.86)	1.64 (0.36–2.44)	<0.001
Serum creatinine (μmol/L)	98 (70–133)	59 (54–72)	0.010
C3 (mg/mL) (mean±SD)	0.41±0.20	0.51±0.18	0.145
Number of positive ANA (%)	72 (96.0)	10 (100)	0.520
Number of positive anti-dsDNA antibodies (%)	58 (77.3)	9 (90.0)	0.357
SLEDAI (mean±SD)	19±5	15±7	0.049
Renal SLEDAI (mean±SD)	11±2	8±4	0.014
Non-renal SLEDAI (mean±SD)	8±5	7±5	0.357
mTOR inhibitors treatment	0	0	NA
Renal histopathology			
Classification			0.001
Class II (%)	0 (0.0)	1 (10.0)	
Class III (%)	9 (12.0)	5 (50.0)	
Class IV (%)	61 (81.3)	2 (20.0)	
Class V (%)	5 (6.7)	2 (20.0)	
Activity index (median; IQR)	8 (6–11)	3 (2–7)	<0.001
Activity index without endocapillary hypercellularity (median; IQR)	7 (4–10)	3 (1–7)	0.001
Cellular/Fibrocellular crescents (median; IQR)	1 (0–2)	0 (0–1)	0.011
Neutrophils/Karyorrhexis (median; IQR)	1 (1–1)	0 (0–0)	<0.001
Fibrinoid necrosis (median; IQR)	0 (0–0)	0 (0–0)	0.403
Hyaline deposits (median; IQR)	1 (0–1)	0 (0–0)	<0.001
Interstitial inflammation (median; IQR)	1 (1–1)	1 (1–1)	0.154
Chronicity index score (median; IQR)	2 (1–4)	1 (0–2)	0.043
Glomerulosclerosis score (median; IQR)	0 (0–1)	0 (0–1)	0.777
Fibrous crescents (median; IQR)	0 (0–0)	0 (0–0.25)	0.853
Tubular atrophy (median; IQR)	1 (1–1)	1 (0.75–1)	0.144
Interstitial fibrosis (median; IQR)	1 (1–1)	1 (1–1)	0.335

Serum creatinine in mg/dL to mol/L, ×88.4.

dsDNA, double stranded DNA; LN, lupus nephritis; mTOR, mammalian target of the rapamycin; NA, not applicable; SLEDAI, SLE Disease Activity Index.

**Figure 3:**
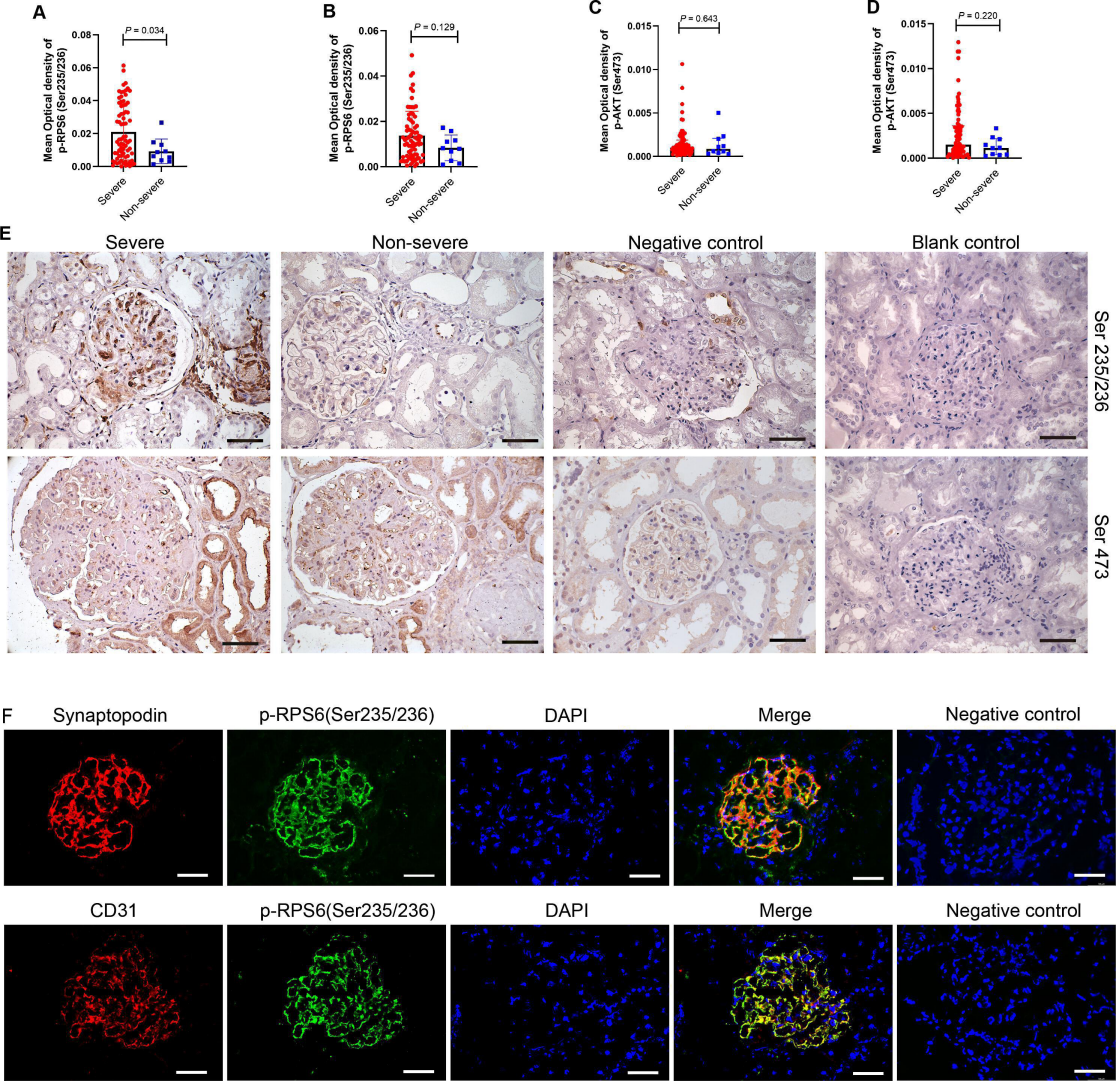
The expression of mTOR complex in kidneys of patients with LN between the non-severe and severe groups. The mean optical density of p-RPS6 (ser235/236) (A-B) and p-AKT (Ser473) (C-D) in the glomeruli and tubulointerstitium between severe and non-severe groups (endocapillary hypercellularity), respectively. (E) Immunohistochemical staining of p-RPS6 (ser235/236) and p-AKT (Ser473) in the glomeruli and tubulointerstitium between severe and non-severe groups, respectively. (F) Colocalisation of p-RPS6 (ser235/236) (green) and synaptopodin (green) (a marker of podocyte), CD31 (red) (a marker of endothelial cells). DAPI, 4′,6-diamidino-2-phenylindole (blue) (a marker of the nucleus); LN, lupus nephritis; mTOR, mammalian target of the rapamycin. Scale bar: 50 µm.

Glomerular mTORC1 activation was significantly higher in the severe group than in the non-severe group (p=0.034; figure 3A and E), and the difference was not significant in the tubulointerstitial area (p=0.129; figure 3B and E). There was no difference in mTORC2 activation between the two groups (glomeruli: p=0.643, tubulointerstitium: p=0.220, figure 3C–E). Furthermore, we found that mTORC1 staining was well colocalised with glomerular endothelial cells and podocytes in patients with LN (figure 3F).

### Correlation analysis of glomerular mTORC1 activation with endothelial-podocyte involvement in patients with LN

**Figure 4:**
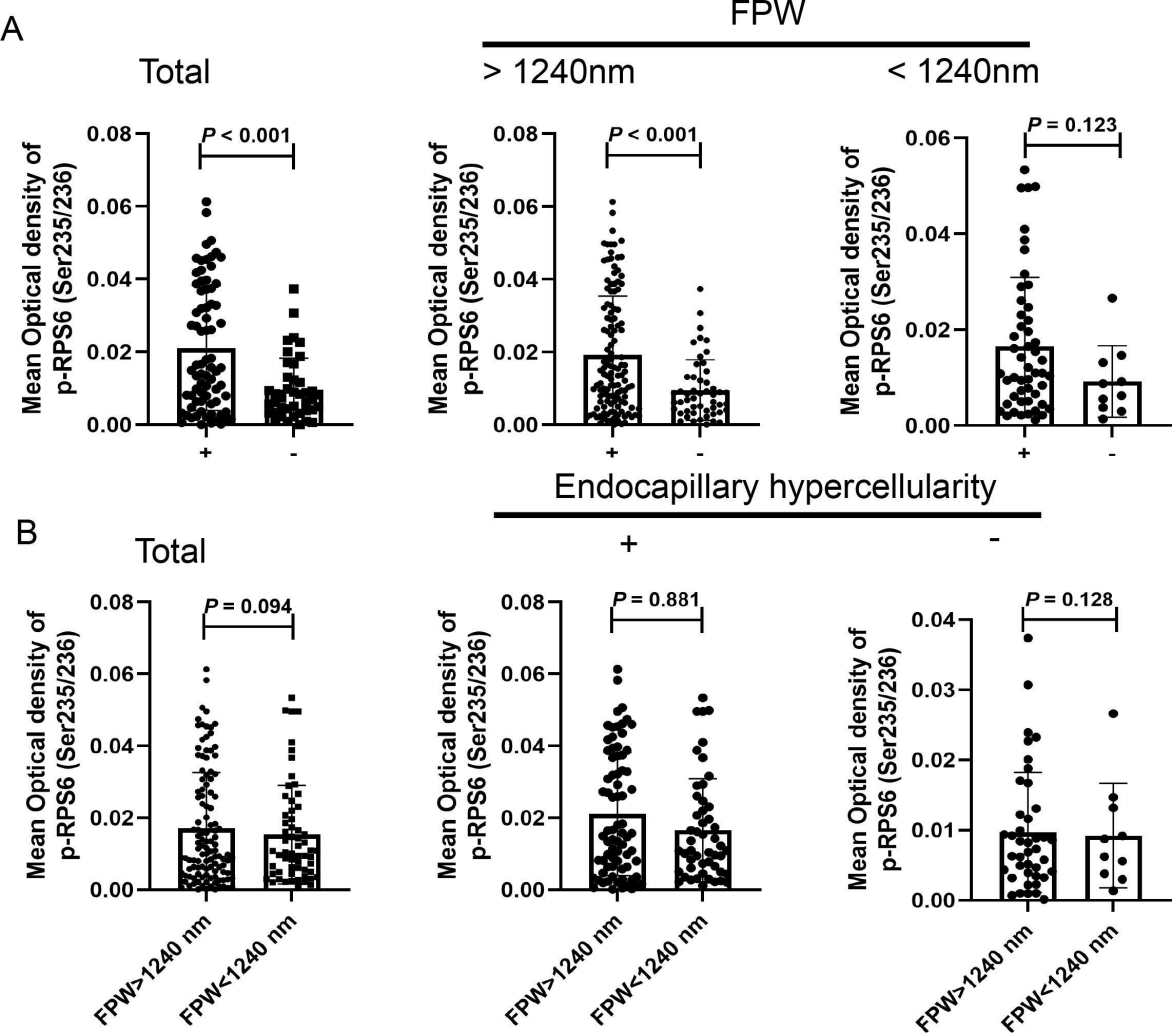
The association of glomerular mTORC1 activation with endocapillary hypercellularity and foot process infusion in patients with LN. (A) Mean optical density of mTORC1 in glomeruli with endocapillary hypercellularity. (B) Mean optical density of mTORC1 in glomeruli with FPW >1240 nm. ‘+’ refers to those patients with endocapillary hypercellularity; ‘−’ refers to those patients without endocapillary hypercellularity. FPW, foot process width; LN, lupus nephritis; mTOR, mammalian target of the rapamycin; mTORC1, mTOR complex 1.

The correlations between mTORC1 activation and endothelial-podocyte involvement were further explored in 176 patients with LN. Among them, endocapillary hypercellularity was significantly positively correlated with mTORC1 activation (r=0.289, p<0.001), although no significant correlation was found between FPW and mTORC1 activation. Moreover, FPW was positively correlated with endocapillary hypercellularity (r=0.234, p=0.002). Patients with LN with more severe endocapillary hypercellularity presented with higher FPW (1415±312 vs 1964±1217, p=0.002) and higher glomerular activation of mTORC1 (p<0.001, figure 4A) than those without it. In particular, in patients with an FPW >1240 nm, the glomerular activation of mTORC1 was significantly higher in patients with endocapillary hypercellularity (p<0.001, figure 4A). No difference was found in glomerular activation of mTORC1 between the groups with FPW >1240 nm and FPW <1240 nm (p=0.094, figure 4B), and there was no association between FPW and mTORC1 activation (r=0.037, p=0.625). We further used the quartiles to divide the FPW into four groups, and mTORC1 activation was significantly higher in the group with FPW at 3/4 than in the group with FPW at 2/4 (p=0.042, online supplemental figure 1).

However, mTORC1 activation in glomerular and tubulointerstitial areas was similar in patients with IgAN with endothelial proliferation and those without endothelial proliferation (p=0.236, online supplemental figure 2A and p=0.379, online supplemental figure 2A–C), although mTORC1 was lightly colocalised with glomerular podocytes and endothelial cells in patients with IgAN (online supplemental figure 2D–E).

We further investigated the renal distribution of the T-cell subsets in patients with LN. CD8^+^ T cells (p=0.0046, online supplemental figure 3A and E), CD4^−^CD8^−^ double-negative T cells (p=0.0052, online supplemental figures 3C, G, H) and T helper (Th)17 cells (p=0.0415, online supplemental figure 3D, I) were significantly higher in the high mTORC1 activation group. The mean optical density of regulatory T (Tregs) cells was similar between the high and low mTORC1 activation groups (p=0.3830, online supplemental figure 3B and F).

## Discussion/Conclusion

The critical role of endothelial-podocyte crosstalk in the development of glomerular lesions through the paracrine process was highlighted in some kidney diseases, including LN.^8–11^ In the current study, we initially explored the mTOR signalling pathway associated with endocapillary hypercellularity and foot process fusion through renal proteomics analysis and verified the significant association of mTORC1 from podocytes to endothelial cells communication based on a well-defined LN cohort.

First, MS-based renal proteomics was applied to discover the difference in signalling pathways between the ‘severe’ endothelial cell and podocyte injury group and the ‘non-severe’ group. GO enrichment analysis revealed that the DEPs of the two groups were primarily involved in some biological processes, including the ‘positive regulation of mTOR signalling’ pathway. Moreover, ‘Ras-related GTPase’ DEPs were both covered in ‘the positive regulation of TOR signalling’ and ‘cellular protein localisation’ of biological process sections. Compelling evidence suggests that the Rag heterodimer (RagA/B and RagC/D) plays a critical role in amino acid signalling to mTORC1 activation by recruiting mTORC1 to the lysosomal membrane,^23^ which could highlight the status of the mTORC1 pathway involved in endothelial cell and podocyte injuries in LN.

Next, our immunohistochemistry work showed that the glomerular activation of mTORC1 was associated with more severe endothelial cell and podocyte injury based on a larger sample. More importantly, mTORC1 staining was found to be well colocalised with glomerular endothelial cells and podocytes, which was consistent with our previous work.^17^ However, no significant associations were found between endothelial proliferation and the activation of mTORC1 in patients with IgAN. Thus, the mechanism of endothelial injury in IgAN and LN might be different.

Last, the correlation analysis from all 176 patients with LN suggested that the patients with more severe endocapillary hypercellularity scores presented with higher FPW and higher glomerular activation of mTORC1. Thus, we proposed that mTORC1 activation might be associated with podocyte-to-endothelial cell communication in LN.

The pathomechanism between mTORC1 activation and endothelial cell-podocyte injury remains unclear. The VEGF-endothelin-1 system has been previously confirmed as a vital process in endothelial-podocyte crosstalk in LN.^7 8 24^ The activation of mTORC1 in podocytes was accompanied by increased VEGF expression in renal tissues in active patients with LN, as well as amplified activation of the mTOR signalling pathway and proliferation of endothelial cells by VEGF stimulation.^25–29^ Nevertheless, the glomerular expression of VEGF was significantly decreased in patients with LN with both TMA changes and FPW ≥1240 nm in our previous results.^7^ The disruption of podocytes by excess activation of mTORC1 may result in decreased VEGF, which further exacerbates endotheliosis.^30 31^ Therefore, mTORC1 activation might be an upstream regulatory signal of podocyte-to-endothelial cell communication, which promotes the development of glomerular lesions in LN. More importantly, our previous study found that glomerular mTORC1 activation was strongly correlated with deteriorative clinicopathological characteristics in patients with LN.^17^ It was proposed that mTOR could develop as a driver of vascular endothelial proliferation and immune activation,^13–16 22^ and excessive activation of mTORC1 in podocytes might result in podocyte loss and subsequent global glomerular sclerosis in mouse models.^32 33^

Moreover, our results indicated that mTOR activation was accompanied by an expansion of T-cell populations, especially diabetes nephropathy (DN) T cells. Previous studies showed that mTORC1 activation in SLE drove the expansion of DN T cells, Th1 cells and Th17 cells and inhibited T-cell lineage specification to Tregs.^34^ DN T cells promote B-cell assistance, leading to the production of pathogenic IgG and the pro-inflammatory cytokines IL-4, IL-17 and interferon-γ,^35^ which might be associated with the increased autoantibody formation involved in glomerular lesions in LN. Therefore, we speculated that the activation of mTOR in T cells played a critical role in the pathogenesis of LN, which needs further exploration.

More interestingly, mTOR inhibition with sirolimus or everolimus could reduce proteinuria and improve kidney function in patients with SLE.^36–38^ Inhibition of mTOR by rapamycin involved mTOR blockade in kidney native cells, reduced necrosis within T cells and DN T cells and expanded Treg populations.^39^ Rational inhibition of mTOR after evaluating the renal activation of mTOR may especially help rescue glomerular endothelial and podocyte injury, which is a potential therapeutic option for LN and deserves further exploration.

Our study had some limitations. Our present study was a retrospective study based on selected phenotypes and a limited evaluation index of podocyte and endothelial cell injury. The proteomics results need to be more widely verified. T-cell activation in the renal tissues was not fully considered in our study, and the comparison with other immune-mediated glomerulonephritis needs to be verified in a larger sample size. The precise role of mTORC1 in endothelial-podocyte crosstalk remains to be further clarified.

In conclusion, glomerular mTORC1 was highly activated in patients with LN with both endocapillary hypercellularity and podocyte injuries, which might be involved in podocytes to endothelial cells communication in LN.

## Data Availability

Data are available in a public, open access repository.
