# Supplementary material for: Glomerular mTORC1 activation was associated with podocytes to endothelial cells communication in lupus nephritis
Source: Lupus Sci Med. 2023 May 5;10(1):e000896. doi: 10.1136/lupus-2023-000896 (PMC10163597; doi:10.1136/lupus-2023-000896)
Supplement: Supplementary data [file lupus-2023-000896supp001.pdf]

Supplementary materials

Supplementary Table 1. The baseline data of 13 lupus nephritis patients for proteomic analysis

Patient	Group	Age/ gender	SLEDAI	Proteinuria, g/day	Scr, μ mol/l	C3, g/L	Anti-nuclear antibody	Anti-dsDNA antibody	Renal Histopathology				
									Classification	AI/CI	Endocapillary hypercellularity	Subendothelial hyaline deposits	FPW, nm
1	1	18/F	14	0.45	78.5	0.20	+	+	IV	3/0	1	1	1267
2	1	42/F	15	0.84	63.0	0.21	+	-	III	6/3	1	1	1328
3	1	69/F	26	6.21	108.9	0.27	+	+	IV	1/0	1	0	2711
4	1	21/M	16	4.35	127.9	0.23	+	+	IV	4/2	1	1	2490
5	1	38/F	16	1.81	67.0	NA	-	-	IV	1/0	1	0	1458
6	1	27/F	2	6.32	48.2	0.44	+	+	III	5/3	1	0	3752
7	1	38/F	18	5.38	133	0.22	+	+	IV	14/4	1	1	1623
8	1	26/M	18	9.30	135	0.37	+	+	IV	6/0	1	0	2379
9	1	34/M	12	0.84	81.1	0.47	+	+	III	4/0	1	1	2277
10	1	40/F	17	4.08	77.0	0.60	+	+	III	6/3	1	0	1529
11	2	13/F	12	0.16	44.0	0.57	+	+	V	0/0	0	0	1216
12	2	42/M	14	3.15	79.5	0.42	+	+	V	0/1	0	0	1172
13	2	22/F	17	9.50	45.9	0.50	-	-	V	2/1	0	1	937

Note: F: female; M: male; Scr: serum creatinine; AI: acute index; CI: chronic index; FPW: foot process width; SLEDAI, systemic lupus erythematosus disease activity index; Anti-dsDNA antibody, anti-double-stranded DNA. Group 1: severe group; group 2: Non-severe group.

Supplementary Table 2. General data of 176 lupus nephritis patients

Index	Description
Clinical Evaluation and Laboratory Assessment	
Sex (male/female), no.	22/154
Age, (yrs), mean ± SD	33±12
SLEDAI, mean ± SD	17±6
Proteinuria (g/24 h), (median; IQR)	2.8(1.4-4.9)
Scr (μmol/L), (median; IQR)	75.6(56.1-109.5)
C3 (g/L), mean ± SD	0.4(0.3-0.6)
Anti-nuclear antibody (+)(%)	170(96.5)
Anti-double-stranded DNA antibody (+)(%)	117(66.5)
Renal Histopathology	
Classification	
Class II (%)	8(4.5)
Class III (%)	31(17.6)
Class IV (%)	100(56.8)
Class V (%)	37(21.0)
Acute index score (median; IQR)	6(3-9)
Endocapillary hypercellularity (median; IQR)	1(0-1)
Cellular/fibrocellular crescents (median; IQR)	1(0-1)
Neutrophils/karyorrhexis (median; IQR)	1(0-1)
Fibrinoid necrosis (median; IQR)	0(0-0)
Hyaline deposits (median; IQR)	0(0-1)
Interstitial Inflammation (median; IQR)	1(1-1)
Chronicity index score (median; IQR)	2(1-3)
Glomerulosclerosis score (median; IQR)	0(0-1)
Fibrous crescents (median; IQR)	0(0-0)
Tubular atrophy (median; IQR)	1(1-1)
Interstitial fibrosis (median; IQR)	1(1-1)
FPW, mean ± SD	1489(1108-2190)

SLEDAI, systemic lupus erythematosus disease activity index; Scr, serum creatinine; IQR, interquartile range; FPW, foot process width.

Supplementary Table 3. The 284 differentially expressed proteins between severe and moderate groups

UniProt ID	Description	Gene Name	P-value	Fold Change
**54 differentially downregulated expressed proteins**				
TTC7B	tetratricopeptide repeat domain 7B(TTC7B)	TTC7B	0.00029316	0.077312192
THEM6	thioesterase superfamily member 6(THEM6)	THEM6	0.00717857	0.131357655
GSTA1	glutathione S-transferase alpha 1(GSTA1)	GSTA1	0.02284454	0.165482199
DMD	dystrophin(DMD)	DMD	0.00778303	0.184909622
UCK1	uridine-cytidine kinase 1(UCK1)	UCK1	0.00136639	0.207814829
KRT6B	keratin 6B(KRT6B)	KRT6B	0.00755151	0.224924313
TMEM38B	transmembrane protein 38B(TMEM38B)	TMEM38B	0.00024419	0.235351148
CLRN3	clarin 3(CLRN3)	CLRN3	0.00038324	0.242921065
CTNNBL1	catenin beta like 1(CTNNBL1)	CTNNBL1	0.00851879	0.256355677
P3H1	prolyl 3-hydroxylase 1(P3H1)	P3H1	0.00455301	0.258077103
MRPS35	mitochondrial ribosomal protein S35(MRPS35)	MRPS35	0.03526684	0.261025053
OXCT1	3-oxoacid CoA-transferase 1(OXCT1)	OXCT1	0.02407023	0.268959303
PDE4DIP	phosphodiesterase 4D interacting protein(PDE4DIP)	PDE4DIP	0.00029979	0.271514012
INHBE	inhibin beta E subunit(INHBE)	INHBE	0.00556809	0.291447357
PYROXD2	pyridine nucleotide-disulphide oxidoreductase domain 2(PYROXD2)	PYROXD2	0.03936044	0.291695467
NUDT12	nudix hydrolase 12(NUDT12)	NUDT12	0.01837059	0.30104546
PARS2	prolyl-tRNA synthetase 2, mitochondrial (putative)(PARS2)	PARS2	0.04929612	0.301740162
CSTF3	cleavage stimulation factor subunit 3(CSTF3)	CSTF3	0.00407399	0.316605668
FBXO22	F-box protein 22(FBXO22)	FBXO22	0.02514262	0.330503658
GNG7	G protein subunit gamma 7(GNG7)	GNG7	0.00050299	0.33273349
SCOC	short coiled-coil protein(SCOC)	SCOC	0.02164541	0.343965189
RAD21	RAD21 cohesin complex component(RAD21)	RAD21	0.02213457	0.354702617
CLCC1	chloride channel CLIC like 1(CLCC1)	CLCC1	0.02342699	0.362421538
PPFIA1	PTPRF interacting protein alpha 1(PPFIA1)	PPFIA1	0.00659459	0.365308791
TMEM177	transmembrane protein 177(TMEM177)	TMEM177	0.01404454	0.367203637
TTYH3	tweety family member 3(TTYH3)	TTYH3	0.01286454	0.367992952
MRPL45	mitochondrial ribosomal protein L45(MRPL45)	MRPL45	0.00325620	0.371773584
AFMID	arylformamidase(AFMID)	AFMID	0.04200421	0.378176384
KIFAP3	kinesin associated protein 3(KIFAP3)	KIFAP3	0.00456015	0.387236768
SIN3A	SIN3 transcription regulator family member A(SIN3A)	SIN3A	0.00342890	0.395549978
FOXRED1	FAD dependent oxidoreductase domain containing 1(FOXRED1)	FOXRED1	0.03087931	0.399400402

Be continued

ZBTB20	zinc finger and BTB domain containing 20(ZBTB20)	ZBTB20	0.03322467	0.403870218
ENPP6	ectonucleotide pyrophosphatase/phosphodiesterase 6(ENPP6)	ENPP6	0.01736699	0.421126942
ACADL	acyl-CoA dehydrogenase, long chain(ACADL)	ACADL	0.03640342	0.424154494
RSF1	remodeling and spacing factor 1(RSF1)	RSF1	0.01952626	0.424562725
TFB1M	transcription factor B1, mitochondrial(TFB1M)	TFB1M	0.04271253	0.427427311
SPRYD4	SPRY domain containing 4(SPRYD4)	SPRYD4	0.02171357	0.428377868
MTMR6	myotubularin related protein 6(MTMR6)	MTMR6	0.00699873	0.438694964
RABL3	RAB, member of RAS oncogene family like 3(RABL3)	RABL3	0.01777239	0.441278593
MRPL28	mitochondrial ribosomal protein L28(MRPL28)	MRPL28	0.01697911	0.443145568
SF3B5	splicing factor 3b subunit 5(SF3B5)	SF3B5	0.02742457	0.443718947
POLDIP2	DNA polymerase delta interacting protein 2(POLDIP2)	POLDIP2	0.01679660	0.447543817
LRRC57	leucine rich repeat containing 57(LRRC57)	LRRC57	0.03429539	0.44867217
NUDT19	nudix hydrolase 19(NUDT19)	NUDT19	0.03731068	0.453037687
TRIM47	tripartite motif containing 47(TRIM47)	TRIM47	0.01837688	0.457032583
PTPMT1	protein tyrosine phosphatase, mitochondrial 1(PTPMT1)	PTPMT1	0.02629364	0.464633517
FGF2	fibroblast growth factor 2(FGF2)	FGF2	0.04937211	0.468396103
IRAK2	interleukin 1 receptor associated kinase 2(IRAK2)	IRAK2	0.04229640	0.471990476
PRKACG	protein kinase cAMP-activated catalytic subunit gamma(PRKACG)	PRKACG	0.04281017	0.472000112
TBRG4	transforming growth factor beta regulator 4(TBRG4)	TBRG4	0.03199141	0.473067785
CBR4	carbonyl reductase 4(CBR4)	CBR4	0.03575183	0.479705487
VAMP2	vesicle associated membrane protein 2(VAMP2)	VAMP2	0.04104667	0.4845826
CIAO2B	family with sequence similarity 96 member B(FAM96B)	CIAO2B	0.02618342	0.488470407
BCKDK	branched chain ketoacid dehydrogenase kinase(BCKDK)	BCKDK	0.02885033	0.498519952
**230 differentially upregulated expressed proteins**				
ALDH3B1	aldehyde dehydrogenase 3 family member B1(ALDH3B1)	ALDH3B1	0.02476132	2.002914706
ALDH3B2	aldehyde dehydrogenase 3 family member B2(ALDH3B2)	ALDH3B2	0.02476132	2.002914706
VWA5A	von Willebrand factor A domain containing	VWA5A	0.03798363	2.003600521

Be continued

	5A(VWA5A)			
DHX36	DEAH-box helicase 36(DHX36)	DHX36	0.04064060	2.004017728
ATG4B	autophagy related 4B cysteine peptidase(ATG4B)	ATG4B	0.02172533	2.009630197
HNRNPUL1	heterogeneous nuclear ribonucleoprotein U like 1(HNRNPUL1)	HNRNPUL1	0.00377826	2.009693419
PLD3	phospholipase D family member 3(PLD3)	PLD3	0.02096436	2.020558245
PFN2	profilin 2(PFN2)	PFN2	0.00919077	2.028756599
GSDMD	gasdermin D(GSDMD)	GSDMD	0.02498522	2.036853327
DYNLT1	dynein light chain Tctex-type 1(DYNLT1)	DYNLT1	0.04644383	2.039420137
APOD	apolipoprotein D(APOD)	APOD	0.02243585	2.046304926
HNRNPLL	heterogeneous nuclear ribonucleoprotein L like(HNRNPLL)	HNRNPLL	0.01263359	2.052981558
THBS1	thrombospondin 1(THBS1)	THBS1	0.00002145	2.073027491
SURF4	surfeit 4(SURF4)	SURF4	0.00164886	2.075621015
SNRPB2	small nuclear ribonucleoprotein polypeptide B2(SNRPB2)	SNRPB2	0.03194093	2.075809865
DAD1	defender against cell death 1(DAD1)	DAD1	0.01532929	2.07686187
SAR1A	secretion associated Ras related GTPase 1A(SAR1A)	SAR1A	0.00108335	2.077583213
GDA	guanine deaminase(GDA)	GDA	0.03799960	2.078854824
HKDC1	hexokinase domain containing 1(HKDC1)	HKDC1	0.01057816	2.0849862
EFHD2	EF-hand domain family member D2(EFHD2)	EFHD2	0.01543222	2.087649691
LGALS1	galectin 1(LGALS1)	LGALS1	0.00343413	2.093452504
NAA50	N(alpha)-acetyltransferase 50, NatE catalytic subunit(NAA50)	NAA50	0.02936376	2.130292774
SERPINB9	serpin family B member 9(SERPINB9)	SERPINB9	0.01628502	2.139430796
TM9SF3	transmembrane 9 superfamily member 3(TM9SF3)	TM9SF3	0.03697617	2.144075132
MAPRE3	microtubule associated protein RP/EB family member 3(MAPRE3)	MAPRE3	0.00835519	2.151476875
TMX4	thioredoxin related transmembrane protein 4(TMX4)	TMX4	0.02909259	2.184902863
SLC35B2	solute carrier family 35 member B2(SLC35B2)	SLC35B2	0.00800823	2.185486383
RCC1	regulator of chromosome condensation 1(RCC1)	RCC1	0.01954603	2.198793721
MCU	mitochondrial calcium uniporter(MCU)	MCU	0.04290235	2.204655135
TWF2	twinfilin actin binding protein 2(TWF2)	TWF2	0.04892754	2.214270152
VAV2	vav guanine nucleotide exchange factor 2(VAV2)	VAV2	0.03777287	2.234336543
RCN2	reticulocalbin 2(RCN2)	RCN2	0.00170138	2.266055216
SRP72	signal recognition particle 72(SRP72)	SRP72	0.00012557	2.269274151

Be continued

IFI30	IFI30, lysosomal thiol reductase(IFI30)	IFI30	0.03974131	2.269828935
CDC73	cell division cycle 73(CDC73)	CDC73	0.02438375	2.270544319
SRP68	signal recognition particle 68(SRP68)	SRP68	0.00049103	2.278253305
RSL1D1	ribosomal L1 domain containing 1(RSL1D1)	RSL1D1	0.04559553	2.289740863
SLC12A6	solute carrier family 12 member 6(SLC12A6)	SLC12A6	0.00229814	2.303296468
SLC12A4	solute carrier family 12 member 4(SLC12A4)	SLC12A4	0.00229814	2.303296468
DFFA	DNA fragmentation factor subunit alpha(DFFA)	DFFA	0.01257109	2.310542937
GNB4	G protein subunit beta 4(GNB4)	GNB4	0.02069002	2.310764999
CRKL	CRK like proto-oncogene, adaptor protein(CRKL)	CRKL	0.02617146	2.342853306
IGF2BP2	insulin like growth factor 2 mRNA binding protein 2(IGF2BP2)	IGF2BP2	0.01449741	2.349553885
TXLNA	taxilin alpha(TXLNA)	TXLNA	0.03192475	2.364080274
BLOC1S4	biogenesis of lysosomal organelles complex 1 subunit 4(BLOC1S4)	BLOC1S4	0.03502529	2.371418642
TGM2	transglutaminase 2(TGM2)	TGM2	0.01902131	2.378156815
IGKV1-8	immunoglobulin kappa variable 1-8(IGKV1-8)	IGKV1-8	0.01750446	2.407530225
FMNL3	formin like 3(FMNL3)	FMNL3	0.01622723	2.42061035
FMNL2	formin like 2(FMNL2)	FMNL2	0.01622723	2.42061035
ATP13A1	ATPase 13A1(ATP13A1)	ATP13A1	0.02376667	2.435083722
PI4K2A	phosphatidylinositol 4-kinase type 2 alpha(PI4K2A)	PI4K2A	0.03384578	2.445719007
COL15A1	collagen type XV alpha 1 chain(COL15A1)	COL15A1	0.01745922	2.453830888
VAMP8	vesicle associated membrane protein 8(VAMP8)	VAMP8	0.00077360	2.455506857
DIAPH1	diaphanous related formin 1(DIAPH1)	DIAPH1	0.01277735	2.483589533
STK3	serine/threonine kinase 3(STK3)	STK3	0.01527802	2.485226568
MAN2A1	mannosidase alpha class 2A member 1(MAN2A1)	MAN2A1	0.02032246	2.491580881
CYRIB	family with sequence similarity 49 member B(FAM49B)	CYRIB	0.04913288	2.492225579
SORT1	sortilin 1(SORT1)	SORT1	0.02942598	2.496640382
SLC35F6	solute carrier family 35 member F6(SLC35F6)	SLC35F6	0.00268841	2.540644189
RCN1	reticulocalbin 1(RCN1)	RCN1	0.04602544	2.543810145
MCTS1	MCTS1, re-initiation and release factor(MCTS1)	MCTS1	0.00593080	2.593394863
RAB35	RAB35, member RAS oncogene family(RAB35)	RAB35	0.00552876	2.598962734

Be continued

TXNDC5	thioredoxin domain containing 5(TXNDC5)	TXNDC5	0.00940224	2.613491011
GGCT	gamma-glutamylcyclotransferase(GGCT)	GGCT	0.00135840	2.647049962
GEMIN5	gem nuclear organelle associated protein 5(GEMIN5)	GEMIN5	0.01480464	2.654477167
BAG3	BCL2 associated athanogene 3(BAG3)	BAG3	0.03452367	2.65757298
ATP2B1	ATPase plasma membrane Ca2+ transporting 1(ATP2B1)	ATP2B1	0.00327735	2.669126958
MAMDC2	MAM domain containing 2(MAMDC2)	MAMDC2	0.02793608	2.684082243
CA4	carbonic anhydrase 4(CA4)	CA4	0.01947327	2.685525522
CCDC6	coiled-coil domain containing 6(CCDC6)	CCDC6	0.03031260	2.687741777
NOP58	NOP58 ribonucleoprotein(NOP58)	NOP58	0.00161103	2.713353466
ADGRE5	adhesion G protein-coupled receptor E5(ADGRE5)	ADGRE5	0.04433741	2.731829865
WARS2	tryptophanyl tRNA synthetase 2, mitochondrial(WARS2)	WARS2	0.03396593	2.737298269
IGFBP7	insulin like growth factor binding protein 7(IGFBP7)	IGFBP7	0.00065796	2.737729991
PLPBP	proline synthetase cotranscribed homolog (bacterial)(PROSC)	PLPBP	0.03264033	2.749460594
IAH1	isoamyl acetate-hydrolyzing esterase 1 homolog(IAH1)	IAH1	0.00740151	2.752799557
RRAGB	Ras related GTP binding B(RRAGB)	RRAGB	0.01741720	2.753260926
RRAGA	Ras related GTP binding A(RRAGA)	RRAGA	0.01741720	2.753260926
NECTIN2	nectin cell adhesion molecule 2(NECTIN2)	NECTIN2	0.04085362	2.762422824
USP47	ubiquitin specific peptidase 47(USP47)	USP47	0.01340378	2.816507645
HLA-DQB1	major histocompatibility complex, class II, DQ beta 1(HLA-DQB1)	HLA-DQB1	0.00074981	2.828034836
SGPL1	sphingosine-1-phosphate lyase 1(SGPL1)	SGPL1	0.04284258	2.836826127
TMSB10	thymosin beta 10(TMSB10)	TMSB10	0.02396017	2.841624317
DDX58	DExD/H-box helicase 58(DDX58)	DDX58	0.03525258	2.864432697
PTGES3	prostaglandin E synthase 3(PTGES3)	PTGES3	0.00022467	2.883425258
LSP1	lymphocyte-specific protein 1(LSP1)	LSP1	0.00897652	2.899681429
PTPN6	protein tyrosine phosphatase, non-receptor type 6(PTPN6)	PTPN6	0.01118396	2.912782062
IGF2R	insulin like growth factor 2 receptor(IGF2R)	IGF2R	0.02533684	2.917016517
MYADM	myeloid associated differentiation marker(MYADM)	MYADM	0.01647572	2.925858479
SDF4	stromal cell derived factor 4(SDF4)	SDF4	0.02785344	2.960812708
SBSPON	somatomedin B and thrombospondin type 1 domain containing(SBSPON)	SBSPON	0.04679579	2.964318931
NHLRC2	NHL repeat containing 2(NHLRC2)	NHLRC2	0.00783092	2.966700666
VCAM1	vascular cell adhesion molecule 1(VCAM1)	VCAM1	0.02554074	2.970817655
TRIP10	thyroid hormone receptor interactor 10(TRIP10)	TRIP10	0.03177997	2.982524829

Be continued

RRAGC	Ras related GTP binding C(RRAGC)	RRAGC	0.01939810	2.987226547
RRAGD	Ras related GTP binding D(RRAGD)	RRAGD	0.01939810	2.987226547
CFI	complement factor I(CFI)	CFI	0.03732764	3.000368147
FKBP11	FK506 binding protein 11(FKBP11)	FKBP11	0.04483646	3.018458537
SERPING1	serpin family G member 1(SERPING1)	SERPING1	0.03380180	3.034320019
MYBBP1A	MYB binding protein 1a(MYBBP1A)	MYBBP1A	0.02313738	3.105991472
ITIH4	inter-alpha-trypsin inhibitor heavy chain family member 4(ITIH4)	ITIH4	0.04232363	3.141084259
TPP2	tripeptidyl peptidase 2(TPP2)	TPP2	0.00349541	3.143233358
STX4	syntaxin 4(STX4)	STX4	0.01129320	3.152955797
ERLEC1	endoplasmic reticulum lectin 1(ERLEC1)	ERLEC1	0.00101779	3.165749748
GBA	glucosylceramidase beta(GBA)	GBA	0.00170098	3.194799966
SUPT6H	SPT6 homolog, histone chaperone(SUPT6H)	SUPT6H	0.04633251	3.248836995
PTPN1	protein tyrosine phosphatase, non-receptor type 1(PTPN1)	PTPN1	0.00993062	3.258257483
GAPVD1	GTPase activating protein and VPS9 domains 1(GAPVD1)	GAPVD1	0.01667479	3.280087674
NRP1	neuropilin 1(NRP1)	NRP1	0.02871458	3.281176757
C7	complement C7(C7)	C7	0.03770100	3.295897988
RER1	retention in endoplasmic reticulum sorting receptor 1(RER1)	RER1	0.00693527	3.415473071
GALC	galactosylceramidase(GALC)	GALC	0.00418345	3.470109987
NCOR1	nuclear receptor corepressor 1(NCOR1)	NCOR1	0.04428310	3.531877781
ABHD6	abhydrolase domain containing 6(ABHD6)	ABHD6	0.04468987	3.640876608
CYP3A5	cytochrome P450 family 3 subfamily A member 5(CYP3A5)	CYP3A5	0.00092985	3.653480429
LRCH4	leucine rich repeats and calponin homology domain containing 4(LRCH4)	LRCH4	0.00938805	3.655330055
STRA6	stimulated by retinoic acid 6(STRA6)	STRA6	0.02651764	3.70156853
LCMT1	leucine carboxyl methyltransferase 1(LCMT1)	LCMT1	0.01499566	3.707627822
UGT3A1	UDP glycosyltransferase family 3 member A1(UGT3A1)	UGT3A1	0.00226652	3.725075756
FABP5	fatty acid binding protein 5(FABP5)	FABP5	0.01752354	3.731182003
DPYSL4	dihydropyrimidinase like 4(DPYSL4)	DPYSL4	0.00438720	3.842024431
ORM2	orosomucoid 2(ORM2)	ORM2	0.03577612	3.87593157
TIAL1	TIA1 cytotoxic granule associated RNA binding protein like 1(TIAL1)	TIAL1	0.01594049	3.890716772
TIA1	TIA1 cytotoxic granule associated RNA binding protein(TIA1)	TIA1	0.01594049	3.890716772
F13A1	coagulation factor XIII A chain(F13A1)	F13A1	0.01334489	4.028664453
GAL3ST1	galactose-3-O-sulfotransferase 1(GAL3ST1)	GAL3ST1	0.00137083	4.119686971

Be continued

SNX6	sorting nexin 6(SNX6)	SNX6	0.04858406	4.135061868
EFL1	elongation factor like GTPase 1(EFL1)	EFL1	0.01399134	4.286568718
MYL1	myosin light chain 1(MYL1)	MYL1	0.02575562	4.298035993
TBC1D1	TBC1 domain family member 1(TBC1D1)	TBC1D1	0.02841964	4.331467192
PPP6C	protein phosphatase 6 catalytic subunit(PPP6C)	PPP6C	0.03538087	4.343703335
CMPK2	cytidine/uridine monophosphate kinase 2(CMPK2)	CMPK2	0.03050324	4.565721038
RASAL1	RAS protein activator like 1(RASAL1)	RASAL1	0.00085441	4.93246446
ACSL4	acyl-CoA synthetase long-chain family member 4(ACSL4)	ACSL4	0.00012933	5.145342571
CD63	CD63 molecule(CD63)	CD63	0.00255891	5.318622818
STING1	transmembrane protein 173(TMEM173)	STING1	0.00590730	5.365128027
FRAS1	Fraser extracellular matrix complex subunit 1(FRAS1)	FRAS1	0.01873524	5.563668225
CTSG	cathepsin G(CTSG)	CTSG	0.02136047	5.743526963
APOC2	apolipoprotein C2(APOC2)	APOC2	0.00342199	5.927918159
TAP2	transporter 2, ATP binding cassette subfamily B member(TAP2)	TAP2	0.01614668	5.948477005
CORO2B	coronin 2B(CORO2B)	CORO2B	0.00606066	6.65787225
TIGAR	TP53 induced glycolysis regulatory phosphatase(TIGAR)	TIGAR	0.00229833	7.638439473
COL3A1	collagen type III alpha 1 chain(COL3A1)	COL3A1	0.02368313	11.29344161
PON2	paraoxonase 2(PON2)	PON2	0.00074868	12.51402878
NNMT	nicotinamide N-methyltransferase(NNMT)	NNMT	0.00048602	13.22618989
POSTN	periostin(POSTN)	POSTN	0.03623033	13.47598519
CD163	CD163 molecule(CD163)	CD163	0.00178565	13.88223144
AMY1A	amylase, alpha 1A (salivary)(AMY1A)	AMY1A	0.02587633	34.28665782
AMY2A	amylase, alpha 2A (pancreatic)(AMY2A)	AMY2A	0.02587633	34.28665782
AMY2B	amylase, alpha 2B (pancreatic)(AMY2B)	AMY2B	0.02587633	34.28665782
PLEK	pleckstrin(PLEK)	PLEK	0.00000000	inf
NEDD8	neural precursor cell expressed, developmentally down-regulated 8(NEDD8)	NEDD8	0.01445884	inf
MCRIP1	MAPK regulated corepressor interacting protein 1(MCRIP1)	MCRIP1	0.00000000	inf
SUPT5H	SPT5 homolog, DSIF elongation factor subunit(SUPT5H)	SUPT5H	0.00000000	inf
B4GAT1	beta-1,4-glucuronyltransferase 1(B4GAT1)	B4GAT1	0.00000000	inf
SELENOF	selenoprotein F(SELENOF)	SELENOF	0.00000000	inf
ATP6AP2	ATPase H+ transporting accessory protein 2(ATP6AP2)	ATP6AP2	0.00000000	inf
YIF1A	Yip1 interacting factor homolog A, membrane trafficking protein(YIF1A)	YIF1A	0.00000002	inf
STBD1	starch binding domain 1(STBD1)	STBD1	0.00000000	inf

Be continued

AGR2	anterior gradient 2, protein disulphide isomerase family member(AGR2)	AGR2	0.00020485	inf
F12	coagulation factor XII(F12)	F12	0.00000139	inf
OAS1	2'-5'-oligoadenylate synthetase 1(OAS1)	OAS1	0.00000895	inf
ANG	angiogenin(ANG)	ANG	0.00160915	inf
C2	complement C2(C2)	C2	0.00000000	inf
SNRPC	small nuclear ribonucleoprotein polypeptide C(SNRPC)	SNRPC	0.00000000	inf
RNASE3	ribonuclease A family member 3(RNASE3)	RNASE3	0.00000000	inf
CD58	CD58 molecule(CD58)	CD58	0.00000000	inf
MRC1	mannose receptor, C type 1(MRC1)	MRC1	0.00000000	inf
NFYA	nuclear transcription factor Y subunit alpha(NFYA)	NFYA	0.00000000	inf
CPOX	coproporphyrinogen oxidase(CPOX)	CPOX	0.00000000	inf
MTHFR	methylenetetrahydrofolate reductase(MTHFR)	MTHFR	0.00000000	inf
NOP2	NOP2 nucleolar protein(NOP2)	NOP2	0.00000000	inf
SERPINB8	serpin family B member 8(SERPINB8)	SERPINB8	0.00000514	inf
ROMO1	reactive oxygen species modulator 1(ROMO1)	ROMO1	0.03231230	inf
HLA-DRB3	major histocompatibility complex, class II, DR beta 3(HLA-DRB3)	HLA-DRB3	0.00000000	inf
CCZ1B	CCZ1 homolog, vacuolar protein trafficking and biogenesis associated(CCZ1)	CCZ1B	0.00000000	inf
CCZ1	CCZ1 homolog, vacuolar protein trafficking and biogenesis associated(CCZ1)	CCZ1	0.00000000	inf
ARHGAP4	Rho GTPase activating protein 4(ARHGAP4)	ARHGAP4	0.00000054	inf
XPC	XPC complex subunit, DNA damage recognition and repair factor(XPC)	XPC	0.00000000	inf
ITIH3	inter-alpha-trypsin inhibitor heavy chain 3(ITIH3)	ITIH3	0.00000000	inf
SNTA1	syntrophin alpha 1(SNTA1)	SNTA1	0.00000000	inf
PKP1	plakophilin 1(PKP1)	PKP1	0.00000000	inf
COG2	component of oligomeric golgi complex 2(COG2)	COG2	0.00000001	inf
RRS1	ribosome biogenesis regulator homolog(RRS1)	RRS1	0.00000000	inf
CYBRD1	cytochrome b reductase 1(CYBRD1)	CYBRD1	0.00003325	inf
TBCEL	tubulin folding cofactor E like(TBCEL)	TBCEL	0.00000000	inf
EXOSC6	exosome component 6(EXOSC6)	EXOSC6	0.00000012	inf
FRY	FRY microtubule binding protein(FRY)	FRY	0.00000005	inf
RALGAPA1	Ral GTPase activating protein catalytic alpha subunit 1(RALGAPA1)	RALGAPA1	0.00000000	inf

Be continued

RCSD1	RCSD domain containing 1(RCSD1)	RCSD1	0.00000000	inf
PLBD1	phospholipase B domain containing 1(PLBD1)	PLBD1	0.00000001	inf
RIPOR1	family with sequence similarity 65 member A(FAM65A)	RIPOR1	0.00000000	inf
SPATC1	spermatogenesis and centriole associated 1(SPATC1)	SPATC1	0.00000963	inf
DHX29	DExH-box helicase 29(DHX29)	DHX29	0.00000000	inf
COMMD7	COMM domain containing 7(COMMD7)	COMMD7	0.00000000	inf
NUDCD3	NudC domain containing 3(NUDCD3)	NUDCD3	0.00000000	inf
SLC39A11	solute carrier family 39 member 11(SLC39A11)	SLC39A11	0.00000000	inf
ARL6IP6	ADP ribosylation factor like GTPase 6 interacting protein 6(ARL6IP6)	ARL6IP6	0.00000000	inf
GIMAP8	GTPase, IMAP family member 8(GIMAP8)	GIMAP8	0.00000000	inf
SH3TC1	SH3 domain and tetratricopeptide repeats 1(SH3TC1)	SH3TC1	0.00000000	inf
ZC3H15	zinc finger CCCH-type containing 15(ZC3H15)	ZC3H15	0.00001642	inf
PPP1R13L	protein phosphatase 1 regulatory subunit 13 like(PPP1R13L)	PPP1R13L	0.00000000	inf
AHCTF1	AT-hook containing transcription factor 1(AHCTF1)	AHCTF1	0.00000000	inf
OVCA2	ovarian tumor suppressor candidate 2(OVCA2)	OVCA2	0.00000001	inf
ANKS1A	ankyrin repeat and sterile alpha motif domain containing 1A(ANKS1A)	ANKS1A	0.00000000	inf
VTI1A	vesicle transport through interaction with t-SNAREs 1A(VTI1A)	VTI1A	0.00000000	inf
PPP1R14B	protein phosphatase 1 regulatory inhibitor subunit 14B(PPP1R14B)	PPP1R14B	0.00000000	inf
ELMO2	engulfment and cell motility 2(ELMO2)	ELMO2	0.00000000	inf
TMEM68	transmembrane protein 68(TMEM68)	TMEM68	0.00000005	inf
SH3GL2	SH3 domain containing GRB2 like 2, endophilin A1(SH3GL2)	SH3GL2	0.00000005	inf
NUP58	nucleoporin 58(NUP58)	NUP58	0.00000000	inf
EMILIN2	elastin microfibril interfacer 2(EMILIN2)	EMILIN2	0.00000000	inf
ZDHHC5	zinc finger DHHC-type containing 5(ZDHHC5)	ZDHHC5	0.00000000	inf
CDC42EP4	CDC42 effector protein 4(CDC42EP4)	CDC42EP4	0.00000000	inf
AGO3	argonaute 3, RISC catalytic component(AGO3)	AGO3	0.00051396	inf
RETN	resistin(RETN)	RETN	0.00000000	inf
LMBRD1	LMBR1 domain containing 1(LMBRD1)	LMBRD1	0.00000038	inf

Be continued

MARCHF5	membrane associated ring-CH-type finger 5(MARCH5)	MARCHF5	0.00000663	inf
TMOD2	tropomodulin 2(TMOD2)	TMOD2	0.00717370	inf
DPM3	dolichyl-phosphate mannosyltransferase subunit 3(DPM3)	DPM3	0.00000078	inf
PISD	phosphatidylserine decarboxylase(PISD)	PISD	0.00000000	inf
ABCF2	ATP binding cassette subfamily F member 2(ABCF2)	ABCF2	0.00000000	inf
TAGLN3	transgelin 3(TAGLN3)	TAGLN3	0.00000000	inf
MBD1	methyl-CpG binding domain protein 1(MBD1)	MBD1	0.00000000	inf
TRMT6	tRNA methyltransferase 6(TRMT6)	TRMT6	0.00000001	inf
NDUFAF1	NADH:ubiquinone oxidoreductase complex assembly factor 1(NDUFAF1)	NDUFAF1	0.00000000	inf
MRPS16	mitochondrial ribosomal protein S16(MRPS16)	MRPS16	0.00000000	inf
TSSC4	tumor suppressing subtransferable candidate 4(TSSC4)	TSSC4	0.00000000	inf
FHOD1	formin homology 2 domain containing 1(FHOD1)	FHOD1	0.01818940	inf
AP1M2	adaptor related protein complex 1 mu 2 subunit(AP1M2)	AP1M2	0.00000000	inf

Supplementary Table 4. GO enrichment terms from differentially expressed proteins

TOTAL			UP			DOWN		
GO-Term	P-value	Protein_Gene	GO-Term	P-value	Protein_Gene	GO-Term	P-value	Protein_Gene
Biological process								
cellular protein localization	0.00000277	Q96ST3:SIN3A,P08962:CD63,Q5VZM2:RRAGB,Q7L523:RRAGA,Q9HB90:RRAGC,Q9NQL2:RRAGD	cellular protein localization	0.00001717	P08962:CD63,Q5VZM2:RRAGB,Q7L523:RRAGA,Q9HB90:RRAGC,Q9NQL2:RRAGD	mitochondrial translational elongation	0.00178357	P82673:MRPS35,Q13084:MRPL28,Q9BRJ2:MRPL45
mucus secretion	0.00021667	O95994:AGR2,Q9BV40:VAMP8	positive regulation of TOR signaling	0.00009639	Q5VZM2:RRAGB,Q7L523:RRAGA,Q9HB90:RRAGC,Q9NQL2:RRAGD	mitochondrial translational termination	0.00191116	P82673:MRPS35,Q13084:MRPL28,Q9BRJ2:MRPL45
positive regulation of epithelial cell apoptotic process	0.00021667	P31483:TIA1,Q99523:SORT1	positive regulation of epithelial cell apoptotic process	0.00013882	P31483:TIA1,Q99523:SORT1	glutamate secretion	0.00262166	P63027:VAMP2,Q13136:PPFIA1
positive regulation of TOR signaling	0.00022659	Q5VZM2:RRAGB,Q7L523:RRAGA,Q9HB90:RRAGC,Q9NQL2:RRAGD	mucus secretion	0.00013882	O95994:AGR2,Q9BV40:VAMP8	positive regulation of chromatin silencing	0.00293734	Q96ST3:SIN3A
platelet degranulation	0.00055982	P08567:PLEK,P08962:CD63,P19652:ORM2,Q06033:ITIH3,Q14624:ITIH4	platelet degranulation	0.00020165	P08567:PLEK,P08962:CD63,P19652:ORM2,Q06033:ITIH3,Q14624:ITIH4	negative regulation of histone H3-K27 acetylation	0.00293734	Q96ST3:SIN3A
alcohol metabolic process	0.00064368	P43353:ALDH3B1,P48448:ALDH3B2	alcohol metabolic process	0.00041322	P43353:ALDH3B1,P48448:ALDH3B2	positive regulation of calcium-dependent cell-cell adhesion	0.00293734	Q92845:KIFAP3
cellular response to amino acid stimulus	0.00119902	Q5VZM2:RRAGB,Q7L523:RRAGA,Q9HB90:RRAGC,Q9NQL2:RRAGD	cellular response to amino acid stimulus	0.00052331	Q5VZM2:RRAGB,Q7L523:RRAGA,Q9HB90:RRAGC,Q9NQL2:RRAGD	carnitine catabolic process	0.00293734	P28330:ACADL
ethanol catabolic process	0.00127482	P43353:ALDH3B1,P48448:ALDH3B2	ethanol catabolic process	0.00082002	P43353:ALDH3B1,P48448:ALDH3B2	somatic diversification of immunoglobulins	0.00293734	Q8WYA6:CTNNBL1
lipid metabolic process	0.00180203	Q6UWR7:ENPP6,O60488:ACSL4,P05090:APOD,P43353:ALDH3B1,P48448:ALDH3B2,Q01469:FABP5	acute-phase response	0.00097630	P19652:ORM2,Q14624:ITIH4,Q86VB7:CD163	cellular ketone body metabolic process	0.00293734	P55809:OXCT1

Liu X, *et al. Lupus Sci Med* 2023; 10:e000896. doi: 10.1136/lupus-2023-000896

post-Golgi vesicle-mediated transport	0.00184886	P63027:VAMP2,Q12846:STX4,Q9BV40:VAMP8	macroautophagy	0.00116359	Q5VZM2:RRAGB,Q7L523:RRAGA,Q9HB90:RRAGC,Q9NQL2:RRAGD,Q9Y4P1:ATG4B	response to methylglyoxal	0.00293734	Q96ST3:SIN3A
molecular_function								
GTPase activating protein binding	0.00127482	Q14C86:GAPVD1,Q8IVF7:FMNL3	GTPase activating protein binding	0.000820018	Q14C86:GAPVD1,Q8IVF7:FMNL3	calcium-activated potassium channel activity	0.00098624	Q9NVV0:TMEM38B,Q9Y217:MTMR6
potassium:chloride symporter activity	0.002104035	Q9UHW9:SLC12A6,Q9UP95:SLC12A4	potassium:chloride symporter activity	0.001356069	Q9UHW9:SLC12A6,Q9UP95:SLC12A4	phosphatidylglycerophosphatase activity	0.00293734	Q8WUK0:PTPMT1
aldehyde dehydrogenase [NAD(P)+] activity	0.002104035	P43353:ALDH3B1,P48448:ALDH3B2	aldehyde dehydrogenase [NAD(P)+] activity	0.001356069	P43353:ALDH3B1,P48448:ALDH3B2	NADPH dehydrogenase (quinone) activity	0.00293734	Q8N4T8:CBR4
SNARE binding	0.003327028	P63027:VAMP2,Q12846:STX4,Q96AJ9:VTI1A,Q9BV40:VAMP8	guanyl-nucleotide exchange factor activity	0.002165464	P52735:VAV2,P86790:CCZ1B,P86791:CCZ1,P98171:ARHGAP4,Q14C86:GAPVD1	NAD(P)H dehydrogenase (quinone) activity	0.00293734	Q8N4T8:CBR4
3-chloroallyl aldehyde dehydrogenase activity	0.004333097	P43353:ALDH3B1,P48448:ALDH3B2	double-stranded RNA binding	0.002445114	O95786:DDX58,P00973:OAS1,Q9H2U1:DHX36,Q9H9G7:AGO3	glycerophosphocholine cholinephosphodiesterase activity	0.00293734	Q6UWR7:ENPP6
double-stranded RNA binding	0.005399926	O95786:DDX58,P00973:OAS1,Q9H2U1:DHX36,Q9H9G7:AGO3	3-chloroallyl aldehyde dehydrogenase activity	0.002803691	P43353:ALDH3B1,P48448:ALDH3B2	arylformamidase activity	0.00293734	Q63HM1:AFMID
guanyl-nucleotide exchange factor activity	0.005583139	P52735:VAV2,P86790:CCZ1B,P86791:CCZ1,P98171:ARHGAP4,Q14C86:GAPVD1	unfolded protein binding	0.005492218	Q15185:PTGES3,Q8IVD9:NUDCD3,Q96DZ1:ERLEC1,Q9Y375:NDUFAF1	NAD+ diphosphatase activity	0.00293734	Q9BQG2:NUDT12
protein heterodimerization activity	0.007127618	O43187:IRAK2,O00267:SUPT5H,Q6GYQ0:RALGAPA1,Q7L523:RRAGA,Q9HB90:RRAGC,Q9NQL2:RRAGD,Q9UNH7:SNX6	polysaccharide binding	0.008475863	O95210:STBD1,Q8IVN8:SBSPON	chloride channel activity	0.00554550	Q96S66:CLCC1,Q9C0H2:TTYH3
unfolded protein binding	0.011803685	Q15185:PTGES3,Q8IVD9:NUDCD3,Q96DZ1:ERLEC1,Q9Y375:NDUFAF1	single-stranded RNA binding	0.008698707	O95786:DDX58,P09234:SNRPC,Q9H9G7:AGO3	proline-tRNA ligase activity	0.00586624	Q7L3T8:PARS2
polysaccharide	0.012972626	O95210:STBD1,Q8IVN8:SB	protein	0.009104582	O00267:SUPT5H,Q6	3-oxoacyl-[acyl-	0.00586624	Q8N4T8:CBR

binding		SPON	heterodimerization activity		GYQ0:RALGAPA1,Q7L523:RRAGA,Q9HB90:RRAGC,Q9NQL2:RRAGD,Q9UNH7:SNX6	carrier-protein] reductase (NADH) activity		4
cellular_component								
Gtr1-Gtr2 GTPase complex	0.00000005	Q5VZM2:RRAGB,Q7L523:RRAGA,Q9HB90:RRAGC,Q9NQL2:RRAGD	Gtr1-Gtr2 GTPase complex	0.00000002	Q5VZM2:RRAGB,Q7L523:RRAGA,Q9HB90:RRAGC,Q9NQL2:RRAGD	mitochondrion	0.00001816	A8MXV4:NUDT19,P28330:ACADL,P55809:OXCT1,P82673:MRPS35,Q13084:MRPL28,Q7L3T8:PARS2,Q8WUK0:PTPMT1,Q8WW59:SPRYD4,Q969Z0:TBRG4,Q96CU9:FOXRED1,Q9BRJ2:MRPL45,Q9Y2S7:POLDIP2
extracellular exosome	0.00000021	O60262:GNG7,P08263:GSTA1,P63027:VAMP2,Q6UWR7:ENPP6,Q8N9N7:LRRC57,Q8NCR9:CLRN3,Q92845:KIFAP3,Q9C0H2:TTYH3,O60488:ACSL4,O75223:GGCT,O75787:ATP6AP2,O94903:PLPBP,P02655:APOC2,P05090:APOD,P08311:CTSG,P08962:CD63,P12724:RNASE3,P19256:CD58,P19652:ORM2,P19961:AMY2B,P20020:ATP2B1,P22748:CA4,P33241:LSP1,P35080:PFN2,P39059:COL15A1,P43353:ALDH3B1,P46109:CRKL,P50452:SERPINB8,P50453:SERPINB9,P54803:GALC,Q01085:TIAL1,Q01469:FABP5,Q06033:ITIH3,Q12846:STX4,Q13835:PKP1,Q14624:ITIH4,Q15185:PTGES3,Q15286:	EGO complex	0.00000009	Q5VZM2:RRAGB,Q7L523:RRAGA,Q9HB90:RRAGC,Q9NQL2:RRAGD	mitochondrial matrix	0.00118176	P28330:ACADL,P55809:OXCT1,Q7L3T8:PARS2,Q8N4T8:CBR4,Q8WVM0:TFB1M

		RAB35,Q15642:TRIP10,Q15843:NEDD8,Q16706:MAN2A1,Q2TAA2:IAH1,Q53TN4:CYBRD1,Q6ZS17:RIPOR1,Q86VX2:COMMD7,Q8IV08:PLD3,Q8N357:SLC35F6,Q8NBS9:TXNDC5,Q8WYP5:AHCTF1,Q92692:NECTIN2,Q96S97:MYADM,Q9BRK5:SDF4,Q9BV23:ABHD6,Q9BV40:VAMP8,Q9GZZ1:NAA50,Q9H2U1:DHX36,Q9HAV0:GNB4,Q9NR31:SAR1A,Q9Y2T3:GDA						
EGO complex	0.00000023	Q5VZM2:RRAGB,Q7L523:RRAGA,Q9HB90:RRAGC,Q9NQL2:RRAGD	extracellular exosome	0.00000011	O60488:ACSL4,O75223:GGCT,O75787:ATP6AP2,O94903:PLPBP,P02655:APOC2,P05090:APOD,P08311:CTSG,P08962:CD63,P12724:RNASE3,P19256:CD58,P19652:ORM2,P19961:AMY2B,P20020:ATP2B1,P22748:CA4,P33241:LSP1,P35080:PFN2,P39059:COL15A1,P43353:ALDH3B1,P46109:CRKL,P50452:SERPINB8,P50453:SERPINB9,P54803:GALC,Q01085:TIAL1,Q01469:FABP5,Q06033:ITIH3,Q12846:STX4,Q13835:PKP1,Q14624:ITIH4,Q15185:PTGES3,Q15286:RAB35,Q15642:TRIP10,Q15843:NEDD8,Q16706:MAN2A1,Q2TAA2:IAH1,Q53TN4:CYBRD1,Q6ZS17:RIPOR1,Q86VX2:COMMD7,Q8IV08:PLD3,Q8N357:SLC	oxidoreductase complex	0.00293734	Q8N4T8:CBR4

					35F6,Q8NBS9:TXNDC5,Q8WYP5:AHCTF1,Q92692:NECTIN2,Q96S97:MYADM,Q9BRK5:SDF4,Q9BV23:ABHD6,Q9BV40:VAMP8,Q9GZZ1:NAA50,Q9H2U1:DHX36,Q9HAV0:GNB4,Q9NR31:SAR1A,Q9Y2T3:GDA			
lysosomal membrane	0.00000710	P08962:CD63,P79483:HLA-DRB3,P86790:CCZ1B,P86791:CCZ1,Q5VZM2:RRAGB,Q7L523:RRAGA,Q8N357:SLC35F6,Q99523:SORT1,Q9BTU6:PI4K2A,Q9BV40:VAMP8,Q9HAV0:GNB4,Q9NUN5:LMBRD1,Q9UP95:SLC12A4,Q9Y6Q5:AP1M2	lysosomal membrane	0.00000051	P08962:CD63,P79483:HLA-DRB3,P86790:CCZ1B,P86791:CCZ1,Q5VZM2:RRAGB,Q7L523:RRAGA,Q8N357:SLC35F6,Q99523:SORT1,Q9BTU6:PI4K2A,Q9BV40:VAMP8,Q9HAV0:GNB4,Q9NUN5:LMBRD1,Q9UP95:SLC12A4,Q9Y6Q5:AP1M2	chloride channel complex	0.00371620	Q96S66:CLCC1,Q9C0H2:TTYH3
condensed nuclear chromosome	0.00011995	Q92845:KIFAP3,P18754:RCC1,Q15050:RRS1,Q9H9G7:AGO3	lysosome	0.00005322	P13284:IFI30,P54803:GALC,Q01085:TIAL1,Q15642:TRIP10,Q5VZM2:RRAGB,Q6P4A8:PLBD1,Q7L523:RRAGA,Q9HB90:RRAGC,Q9NQL2:RRAGD	mitochondrial nucleoid	0.00471984	Q8WVM0:TFB1M,Q9Y2S7:POLDIP2
lysosome	0.00028640	P13284:IFI30,P54803:GALC,Q01085:TIAL1,Q15642:TRIP10,Q5VZM2:RRAGB,Q6P4A8:PLBD1,Q7L523:RRAGA,Q9HB90:RRAGC,Q9NQL2:RRAGD	membrane	0.00006313	O60488:ACSL4,O75376:NCOR1,O76021:RSL1D1,O95210:STBD1,P08567:PLEK,P19256:CD58,P20020:ATP2B1,P22748:CA4,P33241:LSP1,P40222:TXLNA,P50453:SERPINB9,P79483:HLA-DRB3,Q12846:STX4,Q14C86:GAPVD1,Q16706:MAN2A1,Q7Z304:MAMDC2,Q	mitochondrial inner membrane	0.00511300	P82673:MRPS35,Q13084:MRPL28,Q8WUK0:PTPMT1,Q96CU9:FOXRED1,Q9BRJ2:MRPL45

Liu X, *et al. Lupus Sci Med* 2023; 10:e000896. doi: 10.1136/lupus-2023-000896

					8N1S5:SLC39A11,Q8TB61:SLC35B2,Q8TEQ6:GEMIN5,Q8WVV9:HNRNPLL,Q96JJ3:ELMO2,Q99999:GAL3ST1,Q9BRK5:SDF4,Q9BTU6:PI4K2A,Q9BV40:VAMP8,Q9C0B5:ZDHHC5,Q9H9G7:AGO3,Q9HD20:ATP13A1,Q9NUN5:LMBRD1,Q9NYL4:FKBP11,Q9P2X0:DPM3,Q9UP95:SLC12A4,Q9UQ03:CORO2B,Q9Y2X3:NOP58,Q9Y613:FHOD1			
membrane	0.00031198	P63027:VAMP2,Q8N9N7:LRRC57,Q8WYA6:CTNNBL1,Q96S66:CLCC1,O60488:ACSL4,O75376:NCOR1,O76021:RSL1D1,O95210:STBD1,P08567:PLEK,P19256:CD58,P20020:ATP2B1,P22748:CA4,P33241:LSP1,P40222:TXLNA,P50453:SERPINB9,P79483:HLA-DRB3,Q12846:STX4,Q14C86:GAPVD1,Q16706:MAN2A1,Q7Z304:MAMDC2,Q8N1S5:SLC39A11,Q8TB61:SLC35B2,Q8TEQ6:GEMIN5,Q8WVV9:HNRNPLL,Q96JJ3:ELMO2,Q99999:GAL3ST1,Q9BRK5:SDF4,Q9BTU6:PI4K2A,Q9BV40:VAMP8,Q9C0B5:ZDHHC5,Q9H9G7:AGO3,Q9HD20:ATP13A1,Q9NUN5:LMBRD1,Q9NYL4:FKBP11,Q9P2X0:DPM3,Q9UP95:SLC12A4,Q9UQ03:CORO2B,Q9Y2X3:NOP58,Q9Y613:FHOD1	condensed nuclear chromosome	0.00116143	P18754:RCC1,Q15050:RRS1,Q9H9G7:AGO3	peroxisomal matrix	0.00613005	A8MXV4:NUDT19,Q9BQG2:NUDT12
intracellular membrane-bounded organelle	0.00115326	P63027:VAMP2,Q96S66:CLCC1,O95070:YIF1A,P13284:IFI30,P20020:ATP2B1,P86790	intracellular membrane-bounded organelle	0.00119017	O95070:YIF1A,P13284:IFI30,P20020:ATP2B1,P86790:CCZ1B,	intraciliary transport particle	0.00878672	Q92845:KIFAP3

		:CCZ1B,P86791:CCZ1,Q13835:PKP1,Q15642:TRIP10,Q5VZM2:RRAGB,Q7L523:RRAGA,Q86VX2:COMMD7,Q9HB90:RRAGC,Q9HD20:ATP13A1,Q9Y6Q5:AP1M2			P86791:CCZ1,Q13835:PKP1,Q15642:TRIP10,Q5VZM2:RRAGB,Q7L523:RRAGA,Q86VX2:COMMD7,Q9HB90:RRAGC,Q9HD20:ATP13A1,Q9Y6Q5:AP1M2			
mitochondrion	0.00134037	A8MXV4:NUDT19,P28330:ACADL,P55809:OXCT1,P82673:MRPS35,Q13084:MRPL28,Q7L3T8:PARS2,Q8WUK0:PTPMT1,Q8WW59:SPRYD4,Q969Z0:TBRG4,Q96CU9:FOXRED1,Q9BRJ2:MRPL45,Q9Y2S7:POLDIP2,O94903:PLPBP,O95994:AGR2,P00973:OAS1,P36551:CPOX,P54803:GALC,P60602:ROMO1,Q15286:RAB35,Q5EBM0:CMPK2,Q7Z478:DHX29,Q8N357:SLC35F6,Q8ND71:GIMAP8,Q8NE86:MCU,Q9BTU6:PI4K2A,Q9BV23:ABHD6,Q9Y3D3:MRPS16	clathrin-coated endocytic vesicle	0.00280369	Q15286:RAB35,Q9NUN5:LMBRD1	kinesin II complex	0.00878672	Q92845:KIFAP3
secretory granule membrane	0.00257797	P63027:VAMP2,P22748:CA4,Q9BV40:VAMP8	platelet dense granule lumen	0.00473212	Q06033:ITIH3,Q14624:ITIH4	periciliary membrane compartment	0.00878672	Q92845:KIFAP3

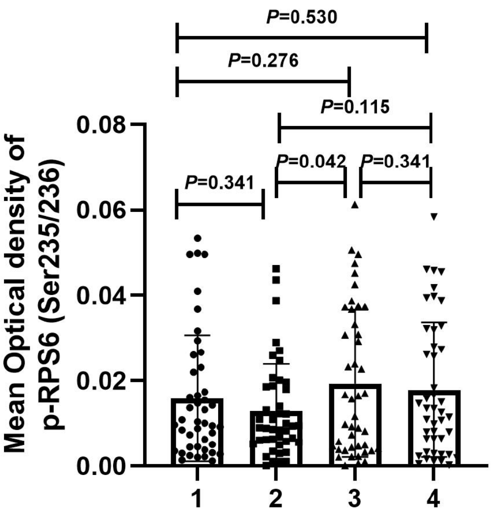

Supplementary Figure 1 The association of mTORC1 activation in glomerular area with FPW. The expression of p-RPS6 (ser235/236) in kidneys of LN patients with different quartiles of FPW. 1, 0 - 1/4; 2, 1/4 - 2/4; 3, 2/4 - 3/4; 4, 3/4 - 1. 1/4, FPW = 1107.7nm; 2/4, FPW = 1488.6nm; 3/4, FPW = 2190.5nm. FPW: foot process width.

Liu X, *et al. Lupus Sci Med* 2023; 10:e000896. doi: 10.1136/lupus-2023-000896

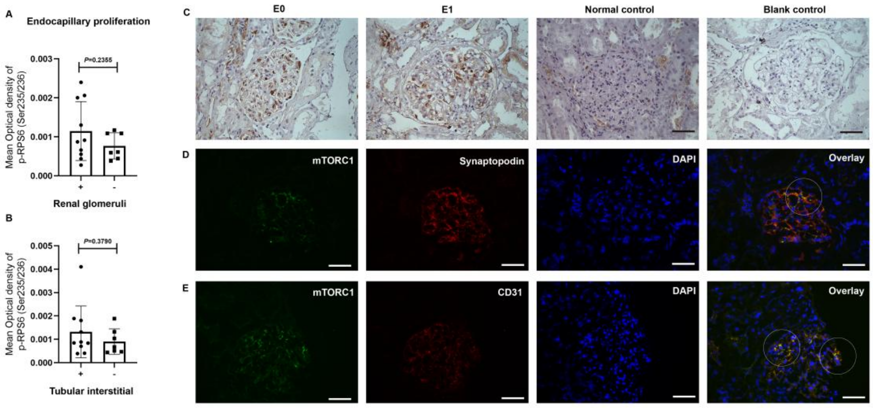

Supplementary Figure 2 The expression of p-RPS6 (ser235/236) in kidneys of IgAN patients. The mean optical density of p-RPS6 (ser235/236) (A-B) and Immunohistochemical staining of p-RPS6 (ser235/236) (C) in the glomeruli and tubulo-interstitium between IgAN with endothelial proliferation and without endothelial proliferation group respectively. (D-E) Colocalization of p-RPS6 (ser235/236) (green) and synaptopodin (green) (marker of podocyte), CD31 (red) (marker of endothelial cells). DAPI, 4′,6-diamidino-2-phenylindole (blue) (marker of nucleus). E: endothelial proliferation. Scale bar: 50 μm.

Liu X, *et al. Lupus Sci Med* 2023; 10:e000896. doi: 10.1136/lupus-2023-000896

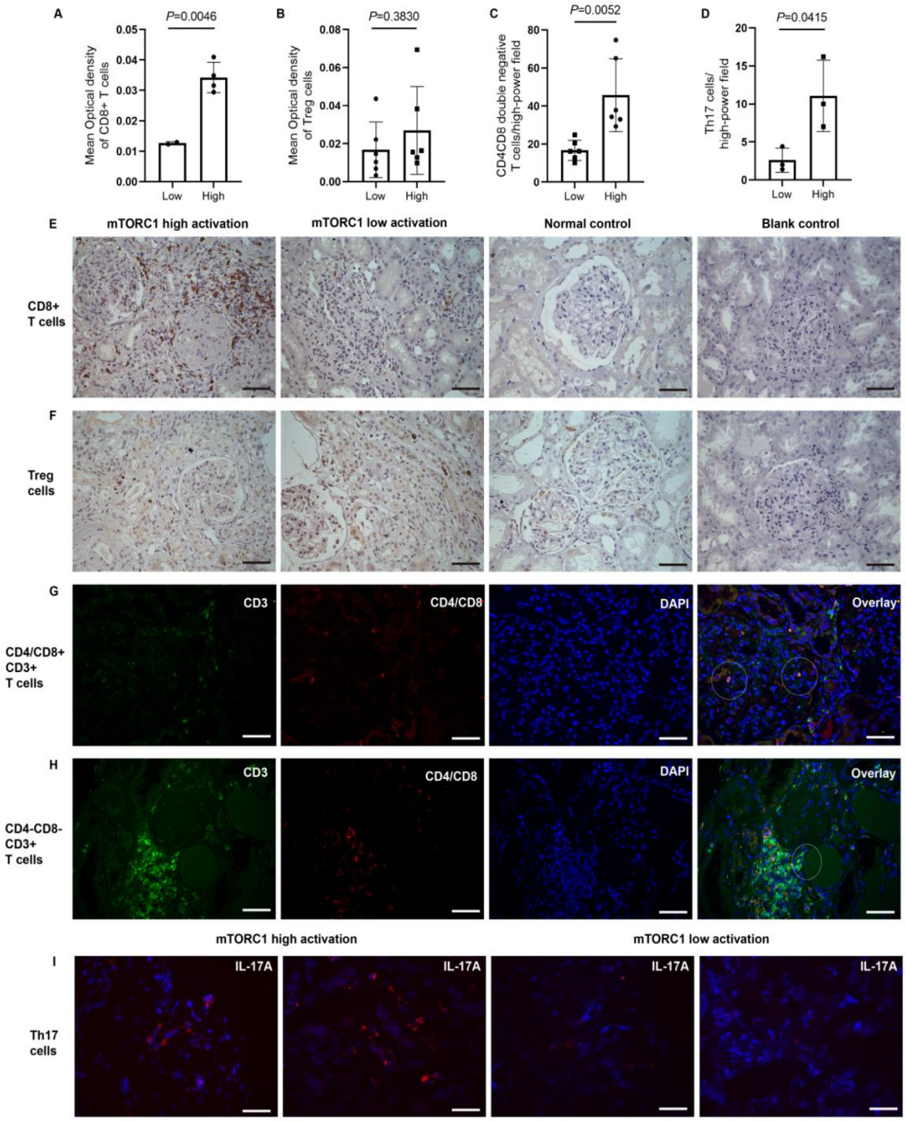

Supplementary Figure 3 The T-cell subset distribution in renal biopsies in LN patinets between mTORC1 high and low activation groups. The mean optical density of CD8+ T cells (A and E) and Treg cells (B and F) in the glomeruli and tubulointerstitium between mTORC1 high and low activation groups, respectively. The number of CD4CD8 double negative T cells (C and G-H, 200×) and Th17 cells

Liu X, *et al. Lupus Sci Med* 2023; 10:e000896. doi: 10.1136/lupus-2023-000896

(D and I, 400×) per high power field. Scale bar: 50 μm.
